# Switching between a computer monitor and virtual reality to reduce the sustained attention decrement

**DOI:** 10.1007/s10055-026-01424-0

**Published:** 2026-08-09

**Authors:** Maxime LeDrew, Adrian Berk Safati, Daniel Smilek

**Affiliations:** Department of Psychology, University of Waterloo, Waterloo, Canada

**Keywords:** Sustained attention, Mind-wandering, Contextual change, Attentional engagement, Performance decrement, Device switching

## Abstract

The present study examined whether changing presentation context can restore sustained attention during monotonous tasks. Participants (N = 164) completed a sustained attention task (i.e., the visual metronome response task) with stimuli presented either on a computer monitor or through a virtual reality (VR) headset. Across two task blocks some participants switched devices (monitor to VR, or VR to monitor), while others used the same device throughout (monitor to monitor, or VR to VR). We observed a sustained attention decrement characterized by increasing response time variability and mind-wandering over time. Participants who switched presentation contexts experienced reductions in mind-wandering compared to those who maintained the same device, demonstrating contextual changes can effectively restore subjective attentional engagement. The type of device switched to (and from) had minimal impact, suggesting the benefit stems from the contextual change rather than the specific presentation mode. Contrary to expectations, overall performance was poorer in VR than on the monitor. However, the effects of switching on task performance were not significant. The results suggest changes in context surrounding a persistent task can be beneficial by temporarily reducing mind-wandering.

**Supplementary Information:**

The online version contains supplementary material available at https://doi.org/10.1007/s10055-026-01424-0.

## Introduction

As immersive virtual reality (VR) environments become increasingly realistic and readily accessible, particularly since the introduction of head-mounted displays (HMDs), they have found a wide range of applications in psychological science. For example, VR has been explored as a therapeutic tool to alleviate the symptoms of various conditions, including attention deficit hyperactivity disorder (for a review see Corrigan et al. 2023), stroke (e.g., Chatterjee et al. 2022), predementia (e.g., Kang et al. 2021) and traumatic brain injury (e.g., De Luca et al. 2023). VR has also been implemented to improve learning in educational contexts (Coban et al. 2022; Makransky & Petersen 2021) and to help individuals hone specific skills (Haque & Srinivasan 2006; Yang et al. 2024). In addition there is a growing body of work utilizing VR as a research tool to explore the basic mechanisms of cognition and related brain processes (Kourtesis et al. 2020; Neguț et al. 2016). Building on this prior work, here we explore whether people can sustain their attention on a task better when it is performed in VR compared to when it is done on a standard computer monitor. More importantly, we also explored whether switching between presentation modes (VR and monitor) can ameliorate the commonly observed sustained attention decrement.

### Sustained attention

Optimal performance in a variety of simple and complex tasks requires that attention remain fully devoted to the task at hand (Petersen & Posner 2012). A common observation is that attention steadily wanes as a function of time on task (Epling et al. 2016; Finkbeiner et al. 2016; Helton & Russell 2011; Mackworth 1948; Pattyn et al. 2008). This temporal decline in attention (i.e., sustained attention decrement; see Langner & Eickhoff (2013) for a review, and Humphrey et al., (2025) for a review from a comparative perspective) has been shown in simple laboratory tasks that require the detection of infrequent target stimuli (e.g., Robertson et al. 1997; Dinges & Powell 1985), as well as more complex tasks like viewing online lecture videos (Risko et al. 2012; Wammes & Smilek 2017). It has been found that as time spent performing a task increases, there is often an increase in subjective feelings of task effortfulness (Kurzban et al. 2013), although in some cases people report decreases in the amount of effort exerted to perform well on their primary task (e.g., Drody et al. 2024). Depending on the specific task and context, as time on task progresses, motivation to perform the task can decrease, while mind-wandering (i.e., internally oriented off-task thought; Brosowsky et al. 2023), boredom (Drody et al. 2024) and off-task media use (Drody et al. 2024, 2025) can increase.

A good example of how task performance, motivation, and mind-wandering change over time on task can be found in a study reported by Brosowsky et al., (2023). In this study participants complete an auditory ‘metronome response task’ (MRT), in which they were required to respond with a button press to a rhythmically presented auditory tone. In such a task, performance is indexed by the variability in response times measured from the onset of the metronome tone to the corresponding button press, whereby poorer performance manifests as increased response time variability (Anderson et al. 2021; Laflamme et al. 2018; Safati et al. 2024; Seli et al. 2013). Brosowsky et al., also included intermittent thought probes asking participants about their momentary level of motivation to perform well on the task, as well as their depth of mind-wandering. They found that over successive blocks of trials, response variability increased (i.e., performance decreased), while motivation decreased, and the depth of mind-wandering increased.

Another example of simultaneous changes in performance and a variety of attention-related subjective experiences is provided by Drody et al., (2024). The primary task performed by participants involved viewing a sequence of letters and detecting the presence of infrequent targets, which were defined as letters that were identical to the letter that was presented one item before in the sequence (a ‘1-back’ task). In one important condition, participants could press a button to display a video above the 1-back stimuli (i.e., to media multitask); video viewing was completely voluntary, and participants were not tested on the contents of the video. Throughout, participants responded to thought probes asking them about the amount of effort they applied to the task, and their level of boredom. As expected, performance on the 1-back task declined over time. Concomitantly, as the task progressed, there was an increase in media multitasking and boredom, as well as a decrease in the actual effort people were willing to devote to the task. Thus, again, as time on task increases, there seems to be a progressive disengagement of attention and effort from the task.

There has been considerable debate regarding the cognitive mechanisms that underlie temporal declines in attention (see Humphrey et al. 2025; Thomson et al. 2015a, b). One class of theories, those designed primarily to explain performance declines in vigilance tasks, suggests that individuals possess an internal ‘pool’ of cognitive resources which may be exhausted by increasing mental exertion as the task progresses, leading to a decrease in task performance over time (Epling et al. 2016; Helton & Russell 2011; Warm et al. 2008). In contrast, another main class of models suggests that, while performing a monotonous task, one becomes progressively under-aroused and thus increasingly disengages attention from the task over time, leading to a steady decrease in task performance (Giambra 1995; Pattyn et al. 2008; Smallwood et al. 2004; Thomson et al. 2015a, b; Zanesco et al. 2025).

Given the negative consequences of attentional declines over time on task, there has been interest in identifying ways that such declines could be reduced or eliminated (see Al-Shargie et al. 2019 for a review). One effective group of interventions targets motivation, with multiple studies showing that increasing participants’ motivation to perform well on a task reduces the magnitude of the sustained attention decrement (Drody et al. 2025; Esterman et al. 2016; Massar et al. 2016; Seli et al. 2019). Another approach involves the introduction of task breaks, which provide a momentary reprieve from the sustained attention decrement (e.g., Arrabito et al. 2015; Colqtjhoun 1959; Mackworth 1948; Ross et al. 2014). Still another potential countermeasure, one that has received relatively little research, is the introduction of new stimuli or variability in task stimuli with the aim of creating dishabituation during otherwise monotonous task contexts (Berlyne et al. 1968; Oken et al. 2006; Thomson et al. 2015a, b). However, the beneficial effects of novelty and stimulus variability on sustained attention performance have been disputed (e.g., Head & Helton 2012; Helton & Russell 2012). Notably, even where increasing stimulus variability has been associated with a shallower temporal decline, overall performance has at times been poorer in the more variable condition (Thomson et al. 2015a, b).

### Immersive virtual reality and sustained attention

This brings us to consider how sustained attention and performance might be influenced when stimuli are presented in an immersive virtual reality (VR) environment. Immersive VR environments have been shown to elicit high ratings of ‘presence’ (Slater & Usoh 1993), whereby participants feel as though they are actually embedded within the virtual environment. For individuals inexperienced with VR technology, completing a task within immersive VR may be experienced as novel and interesting, raising the possibility that VR might increase their attentional engagement with the task at hand relative to completing the task on a standard 2D monitor. This heightened engagement may result in a reduced sustained attention decrement when the task is completed in VR than when it is completed on a more traditional desktop 2D monitor. In contrast, researchers have also raised the possibility that the novel experience of the VR setting might impose added cognitive processing demands, a phenomenon referred to as the “novelty effect” (Miguel-Alonso et al. 2024). This novelty effect might lead users to attend more to the various unfamiliar aspects of the virtual environment (i.e., the 3D nature of the space and the quality of the graphics) and less to the focal task to be performed in the environment (Miguel-Alonso et al. 2024). It is conceivable that such a novelty effect could increase the sustained attention decrement when an attention task is completed in VR compared to a computer screen.

Studies that have compared performance in tasks that require sustained attention across modes of presentation (i.e., 2D computer monitor versus immersive VR) have led to mixed conclusions. Some evidence suggests that people can sustain attention better in VR than when the task stimuli are presented on a 2D monitor. For example, Li et al., (2020) used a spatial-cuing task in which participants were presented with a spatial cue followed by either a target (i.e., one type of fish) or distractor item (i.e., another type of fish). Participants were required to respond if a target stimulus was presented, and to withhold responses if a distractor stimulus was presented. Overall, reaction times were slower and accuracy lower when the task was completed using a 2D monitor than when it was done using the VR device. In contrast, other studies support the opposite conclusion. Baertsch et al., (2023) showed that detection of the presence of a target letter in a string of letters was more accurate and faster when the task was performed on a computer monitor than a head-mounted display. Post-task subjective reports of participants were in line with these behavioral effects, indicating that the task was subjectively easier when done on the computer monitor than on the head-mounted display.

Still other studies have failed to find any difference in attention-related performance across 2D and VR environments. Rupp et al., (2019) had participants complete a vigilance task presented on a 2D computer monitor or via a head-mounted VR device. The vigilance task involved the sequential presentation of shapes, and the participants were required to respond to a predetermined target shape while withholding responses to the other two shapes. The results showed that performance in the vigilance task (in terms of both reaction time and accuracy) was roughly equivalent between the two viewing conditions. Along similar lines, Aksoy et al., (2021) had participants complete a 1-back and a 2-back task, which involved viewing a sequence of letters and detecting target items defined by a match to either the prior item (1-back) or the one 2 items back (2-back) in the sequence. The tasks were completed either on a 2D monitor or in a VR headset, and no difference was found between viewing conditions in both accuracy of target detection and reaction times to targets.

While these prior studies constitute an important initial foray into the impact of immersive VR environments on sustained attention, several critical issues remain unaddressed. First, previous analysis have focused primarily on the overall difference in attention-related performance across VR and 2D displays, but have not clearly delineated the temporal patterns in performance and attention that define the sustained attention decrement. Second, the extant studies have not directly indexed internal inattentive states, particularly as they manifest in the form of mind-wandering. Third, the comparison of attention and performance across VR and 2D monitor display conditions presents a unique opportunity to switch the viewing context within participants in a substantive way (VR to Monitor; Monitor to VR), while holding the fundamental attention task constant. Although to our knowledge this has not been examined directly (cf. Huang et al. 2021), such switches in viewing context could reveal whether changing visual contexts (relative to maintaining the same context) can ameliorate the sustained attention decrement even though the task remains the same. While context switches could be implemented in other ways (such as having participants move between two rooms), we were particularly interested in a potential use of VR technology to achieve a context switch; we also assumed that shifting between VR and Monitor might be an easy way to control for extraneous factors (e.g., the effects of walking when switching contexts) and that the change between VR and 2D Monitor viewing may be particularly stark, thus maximizing the opportunity to find effects of switching contexts.

### The present study

In the present study we explored how sustained attention performance and mind-wandering can be influenced by task context (i.e., computer monitor and VR), even when the task itself remains the same. Specifically, we aimed to 1) compare sustained attention performance and mind-wandering in the same visual task presented on a computer screen versus when presented on a VR HMD, and 2) investigate whether mind-wandering can be reduced and performance restored on a sustained attention task by switching to a distinct presentation format (i.e., context). To address these aims, participants were introduced to a visual version of the metronome response task (VMRT; Laflamme et al. 2018), on either a computer monitor or a VR HMD, which involved providing responses in synchrony with the rhythmic appearance of a target object. Throughout this task, participants were occasionally presented with a thought probe evaluating subjective mind-wandering experiences. Halfway through the experimental session, after participants completed a block of trials on the initial device, one group of participants switched to a device of a different type (Monitor to VR or VR to Monitor), while the other group transitioned to a second device of the same type (Monitor to Monitor, VR to VR). Participants then completed the VMRT on their newly assigned device. In all conditions participants experienced a brief break between the two halves of the experiment since the device swap incurs a break to ensure that the new device is functioning correctly and comfortably; we attempted to equate the breaks between conditions since breaks are known to influence sustained attention (Arrabito et al. 2015; Colqtjhoun 1959; Ross et al. 2014).

We expected several main outcomes. First, given the novel experience and claimed engagingness of VR, we hypothesized that response variability and mind-wandering would be lower when the task was completed in VR than on the computer monitor, and that these indices of inattention would increase less over time-on-task with VR than with the monitor (*Hypothesis 1*). Second, we expected that participants switching to a distinct device should experience greater attention restoration (i.e., reduction in response variability and mind-wandering) following the switch than participants continuing the experiment with the same device (*Hypothesis 2*). Finally, we hypothesized that participants transitioning from the two-dimensional monitor to VR should experience greater attention restoration following the device switch than participants transitioning from VR to the monitor (*Hypothesis 3*).

## Methods

Our research was approved by the Office of Research Ethics at the University of Waterloo (ORE#47,194) and performed in accordance with the principles stated in the Declaration of Helsinki. Informed consent was obtained from all research participants. The deidentified experimental data, analysis code, and experimental materials are available in an OSF repository.

https://osf.io/qymw3/?view_only=ebeac74209f845baa0b52d0aa1ea31be.

### Participants

Participants included 168 undergraduate students, who were recruited from a pool of undergraduate students taking psychology courses. The study took place in-person, and participants were provided course credit in exchange for 45 min of participation. Each participant was randomly assigned to one of four groups depending on the order of the devices they were to use: Monitor to Monitor (M-M), VR to VR (VR-VR), Monitor to VR (M-VR), and VR to Monitor (VR-M). Data from two participants were omitted from analysis because they discontinued the study, and data from another two participants were removed due to a failure to provide a response for over 60% of the trials in at least one of the blocks. After the foregoing removals, 164 participants were included in the analysis, distributed as follows among groups: M-M (n = 42), VR-VR (n = 42), M-VR (n = 41), VR-M (n = 39).

### Materials

#### Visual metronome response task (VMRT)

**Fig. 1:**
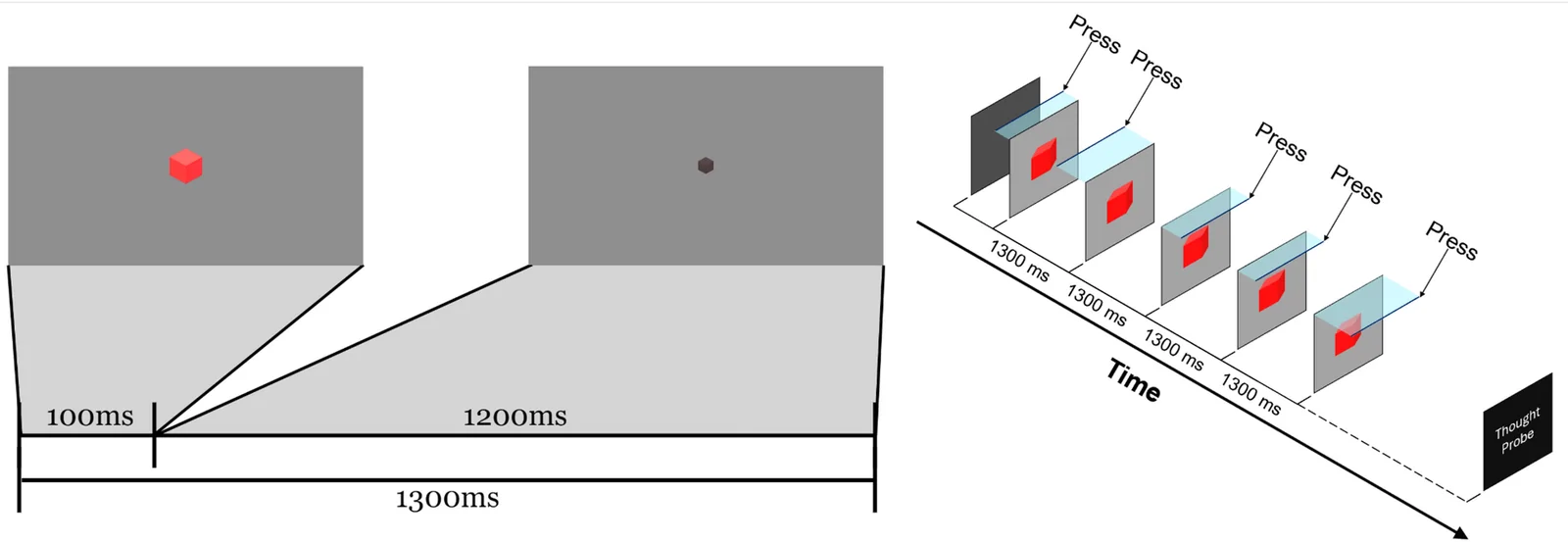
VMRT experimental sequence. Note participants were instructed to press the spacebar in sync with the appearance of the saturated red cube, (i.e., anticipating not reacting to its appearance). In each trial after a brief 100 ms appearance the red cube would shrink and desaturate for 1200 ms serving as a fixation point for the appearance of the cube in subsequent trials

We used a variant of the visual metronome response task (VMRT) (Laflamme et al. 2018) that presented a rhythmically pulsing red cube which alternated between a large, saturated state, and a small, less saturated state. The background was uniformly gray. As seen in Fig. 1, on each trial, the saturated cube would peak in size and saturation for 100 ms before shrinking and fading into a less saturated but still visible state for 1200 ms. The small, less saturated version of the cube served as a fixation point. Participants were instructed to press the keyboard space bar in sync with the moment at which the cube first reached its maximal size and saturation. Response times for each trial were measured as the time between the first moment of maximal size and saturation of the cube and the corresponding spacebar press. Trials without responses were excluded from the analysis. Importantly, attention-related performance was indexed by computing response time variability across discrete windows of five trials. The windows did not include trials that overlapped with those in other windows. For these response time variability (RTv) scores, greater variability reflects an inability to press the space bar in sync with the presentation of the saturated cube, which reflects poorer performance on the task. Critically, across numerous studies, increased response variability has been associated with greater task inattention (e.g., mind-wandering; Anderson et al. 2021; Laflamme et al. 2018; Safati et al. 2024; Seli et al. 2013). A natural logarithm transformation was applied to reduce the positive skew in the data distribution as is common practice in analyses of the MRT (Anderson et al. 2021; Laflamme et al. 2018; Safati et al. 2024; Seli et al. 2013).

#### Thought probes

Throughout the experiment, participants were occasionally presented with thought probes evaluating their subjective experiences of mind-wandering. These thought probes asked, “Since the previous probe, how much did you find yourself thinking about something other than the task at hand?” Participants responded by moving a slider with an integer scale ranging from 0 to 100, such that a value of 0, labeled “*Not at all*”, represented a complete lack of mind-wandering experiences, and the value of 100, labeled “*Almost entirely*”, represented near total engagement in mind-wandering.

### Apparatus

#### 2D Monitor hardware configuration

**Fig. 2:**
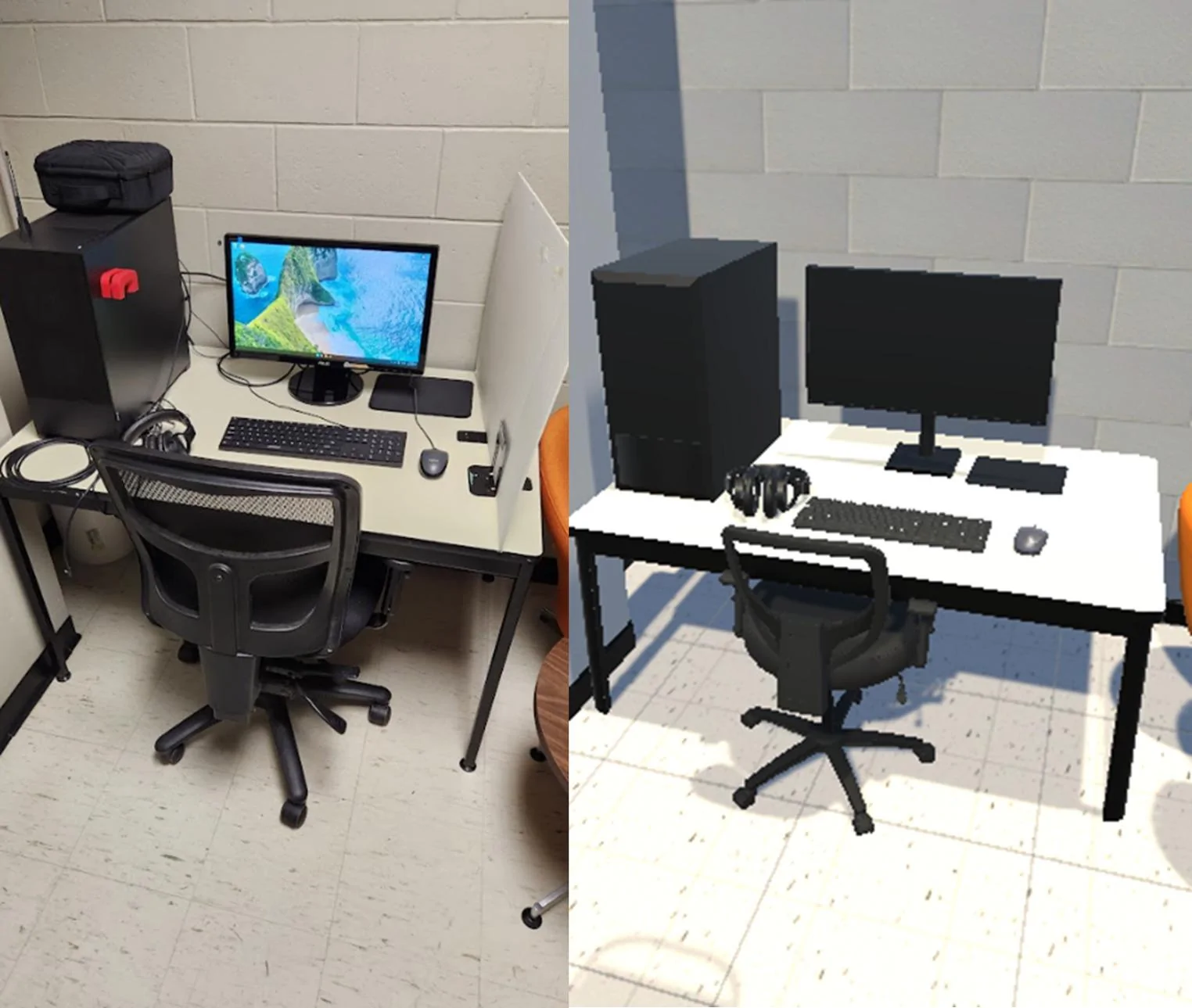
Physical and virtual environments for experimental session. Note the virtual environment (right) was designed to simulate the testing environment of the real world (left)

The 2D display used in this experiment was a Philips 223V5LSB2 Monitor, with a refresh rate of 60 Hz. When in use, the VMRT was presented in borderless full-screen mode, thereby occupying the entire area of the screen. The monitor rested on the desk directly in front of each participant, though the viewing angle varied as a result of height differences among the participants. A photo of the laboratory set-up is shown on the left side of Fig. 2.

#### 2D monitor software configuration

For the monitor condition, the metronome response task was coded in PsychoPy-2024.2.4. Participants used the spacebar of the computer keyboard to advance through the instructions, provide responses to the metronome stimuli, and “lock in” a response to the thought probes. The thought probe responses were provided by using the mouse scroll wheel to move a red “response marker” along the horizontal slider bar, with a downwards scrolling motion moving the marker to the left (i.e. less mind-wandering, labelled “not at all”), and upwards scrolling motions moving the marker to the right (more mind-wandering, labelled “almost entirely”). The response marker was initially positioned in the centre of the thought probe slider, and subsequently appeared wherever participants last responded. This was done to reduce task complexity by minimizing scrolling between consecutive thought probe responses. The scroll wheel was chosen to simplify interaction with the thought-probe slider as much as possible, as the mouse pointer was hidden from the participant’s view in both conditions.

#### 3D HMD hardware configuration

The HMD used in this experiment was a Meta Quest 2 connected to the computer by a standard USB-A to USB-C Cable. The refresh rate was 90 Hz and the brightness settings were tuned to match the perceived brightness of the room in reality.

#### 3D HMD software configuration

The 3D environment within the headset (shown in the right panel of Fig. 2) was designed in Unity 2022.3.40f1 with the OpenXR Unity package to be approximately identical to the real-world laboratory. Each station contained a version of the environment with a “player” origin point equivalent to their actual position in the room. This was done to ensure that the peripheral aspects of participants’ environment remained roughly the same between VR and reality regardless of their position in the room. The VMRT was displayed on a monitor within the virtual environment and was stretched to the borders of this monitor as in the 2D display. The metronome response task in VR was coded in Visual Studio 2022, and controlled identically to the VMRT created in PsychoPy for the 2D Monitor condition. That is, the spacebar was used to respond to the stimuli of the VMRT, advance through instruction screens, and “lock in” a response to the thought probe. Once again, the scroll wheel of the mouse was used to adjust the position of the thought probe response marker, initially appearing at a value of “51” and subsequently appearing at the value previously provided by the participant. Before beginning the VR component of the task, each participant’s left hand was positioned over the spacebar, and each participant’s right hand was positioned above the mouse to eliminate the complexity of a tactile search for these input methods. The coded visual MRT was then assigned to a Canvas object in unity, which in turn was used as a texture that could be projected onto the monitor object at the user’s virtual desk to create the illusion of a functional monitor screen.

### Procedure

Participants were randomly assigned to one of four conditions which determined the device they would use in the first and second blocks. Participants using VR had their inter-pupillary distance (IPD) measured using a pupillometer before beginning the experiment. Using this measurement, the station HMD’s IPD settings were adjusted.

Participants were provided with a short practice session on their initial device, during which 60 trials of the VMRT were presented at the same pace as the experimental task alongside a running count of the number of correct inputs (labeled “hits”). At trials 20 and 40 in the practice block, the task was interrupted, and a thought probe was displayed with instructions on how to interact with the scale. Upon completing the practice trials, participants were instructed to begin the experimental trials by pressing the space bar when they were ready to begin the primary task.

During the primary task, each participant completed two blocks of the VMRT, with each block consisting of 504 trials. Within each block the VMRT was interrupted five times with a thought probe evaluating participants’ subjective experiences of mind-wandering, with one additional thought probe appearing after the final trial. Once the first block had been completed, participants received a prompt instructing them to transition to their second device. While some participants continued using the same 2D monitor or HMD as in the first block, others switched to the alternative device. To partly standardize the task break duration between participants switching and not switching devices, a minimum 60-s waiting period was imposed between blocks. Following the break, participants completed the second block of the VMRT.

### Data analysis

Mixed effects models were developed using the *lme4* package (Bates et al. 2014) in R version 4.5.0 (R Core Team 2024). The *performance* package (Lüdecke et al. 2021) was used to verify model assumptions and to compare random effects structures via AIC, selecting the model with the lowest AIC as optimal. Planned contrasts were conducted using the *emmeans* package (Lenth 2023). The significance for the ANOVA results presented in Table 3 were tested using the Satterthwaite approximation for degrees of freedom with the *lmerTest* package (Kuznetsova et al. 2017). Effect sizes for fixed effects were quantified using partial eta-squared calculated using default settings from the *effectsize* package (Ben-Shachar et al. 2020). To facilitate interpretation, we standardized the estimated mean differences by dividing by the residual standard deviation from each model, yielding effect sizes analogous to Cohen’s *d.*

Model summaries, including formulas, the number of observations, performance indicators, and ANOVA results for the predictors can be found in Table 3. Model coefficients and as needed, their reverse transformed interpretations can be found in Table 4. The dependent measure of Model 1.1 and Model 2.1 is task performance as indexed by response time variability (RTV), while Model 1.2 and Model 2.2 examine mind-wandering reports. Time on task in the models is indexed by either the “segment” of the block in which the trials occurred (Model 1.1 and Model 2.1) or by the “probe” sequence number within the block (Model 1.2 and Model 2.2), and these predictors were grand mean centered. The fixed effects structure of the models was specified to enable testing of our hypothesis. We developed random effects structures by fitting theoretically motivated random slopes for time-varying within-subject factors that could capture individual differences in vigilance trajectories. For each model, we tested whether random slopes for segment (Models 1.1 and 2.1), probe (Models 1.2 and 2.2), and block (Models 2.1 and 2.2) significantly improved model fit compared to random-intercept-only alternatives. Models that converged were compared using likelihood ratio tests and AIC values (Table S1).

Model 1.1 uses data from the first block of the task to examine how response time variability was influenced by task condition {Monitor, or VR}, or time-on-task as indexed by the block segment, and the interaction between these factors. Response time variability scores were calculated as the variance of response times across discrete successive windows of 5 trials, with each window in the series representing the local variability in responses. The model included random intercepts and random slopes for block segment, allowing both baseline response variability and the rate of temporal change to vary across participants.

Model 1.2 included data from the first block of the task to examine how mind-wandering reports were influenced by task condition {Monitor, or VR}, or time-on-task as indexed by the probe number, and the interaction between these factors. The model included random intercepts and random slopes for the probe number, allowing both baseline mind-wandering and the rate of temporal change to vary across participants.

Model 2.1 made use of data from both blocks to examine how response time variability scores were influenced by group {M-M, M-VR, VR-M, VR-VR}, the task block {Block 1, Block2}, and time within blocks indexed by the block segment, and the interactions between these factors. With six mind-wandering probes distributed at equal distances throughout each block, the block segment reflected the six trial periods preceding each probe. This categorization was done to facilitate comparisons between Segment 6 of Block 1, with Segment 1 of Block 2. As with Model 1.1 we included an additional factor to control for potential differences in the variability of stimulus presentation. The model included random intercepts and random slopes for block, allowing both baseline response variability and the effect of switching between blocks to vary across participants.

Model 2.2 used data from both blocks to examine how mind-wandering reports were influenced by group {M-M, M-VR, VR-M, VR-VR}, the task block {Block 1, Block 2}, and time within blocks as indexed by the block probe number, and the interactions between these factors. The model included random intercepts and random slopes for both the probe and block, which meant that baseline mind-wandering, temporal trajectories, and block effects varied across participants.

To address potential technical confounds, we conducted diagnostic analyses retrospectively examining stimulus presentation timing and response recording precision between the Monitor and VR setups. These diagnostics (detailed under "Diagnostics" within the R analysis markdown file in the OSF repository) revealed comparable measurement precision across conditions, making it unlikely that technical factors account for the observed behavioural differences.

## Results

Descriptive statistics for response time variability and mind-wandering reports across experimental groups and blocks are presented in Tables 1 and 2 respectively. In Block 1, before any device switching occurred, participants in monitor-based conditions (M-M and M-VR combined) showed mean response time variability of 21,606.25 ms (*SD* = 49,126.06) or 8.89 (*SD* = 1.39) following transformation, while those in VR-based conditions (VR-M and VR-VR combined) showed mean variability of 29,507.43 ms (*SD* = 54,491.57) or 9.32 (*SD* = 1.38) following transformation. Mind-wandering reports in Block 1 averaged 58.57 (*SD* = 25.73) for monitor-based conditions and 56.08 (*SD* = 29.95) for VR-based conditions. The pattern of changes across blocks varied by experimental group, as detailed in the analyses below.

**Table 1:** Descriptive statistics for response time variability as a function of group in the original scale (ms) and following LN transformation

Group	Block	n	Mean	SD	Skewness	Kurtosis
Original Scale						
M-M	Block 1	42	20,779.76	45,983.07	4.60	27.23
M-M	Block 2	42	23,856.27	49,863.49	4.24	24.31
M-VR	Block 1	41	22,454.86	52,148.80	4.37	23.73
M-VR	Block 2	41	25,920.98	48,955.85	3.96	21.15
VR-M	Block 1	39	29,284.43	51,400.61	3.41	16.43
VR-M	Block 2	39	22,965.31	45,602.60	4.50	27.94
VR-VR	Block 1	42	29,716.07	57,238.39	4.01	21.79
VR-VR	Block 2	42	30,533.64	54,587.75	3.73	19.56
LN transformed for analysis						
M-M	Block 1	42	8.89	1.37	0.31	3.36
M-M	Block 2	42	8.99	1.43	0.26	3.14
M-VR	Block 1	41	8.88	1.41	0.30	3.46
M-VR	Block 2	41	9.22	1.35	-0.01	4.35
VR-M	Block 1	39	9.34	1.37	0.11	3.04
VR-M	Block 2	39	9.07	1.37	0.15	3.13
VR-VR	Block 1	42	9.30	1.40	0.13	3.06
VR-VR	Block 2	42	9.37	1.40	0.05	2.90

**Table 2:** Descriptive statistics for mind-wandering reports (0–100 scale) as a function of group and block

Group	Block	n	Mean	SD	Skewness	Kurtosis
M-M	Block 1	42	62.12	26.66	-0.27	2.12
M-M	Block 2	42	78.60	23.10	-0.96	3.09
M-VR	Block 1	41	54.94	24.28	-0.28	2.31
M-VR	Block 2	41	59.80	25.71	-0.37	2.18
VR-M	Block 1	39	55.85	29.52	-0.27	2.04
VR-M	Block 2	39	64.32	26.41	-0.56	2.45
VR-VR	Block 1	42	56.29	30.39	-0.27	1.83
VR-VR	Block 2	42	70.83	28.30	-0.78	2.52

### Monitor versus virtual reality

**Fig. 3:**
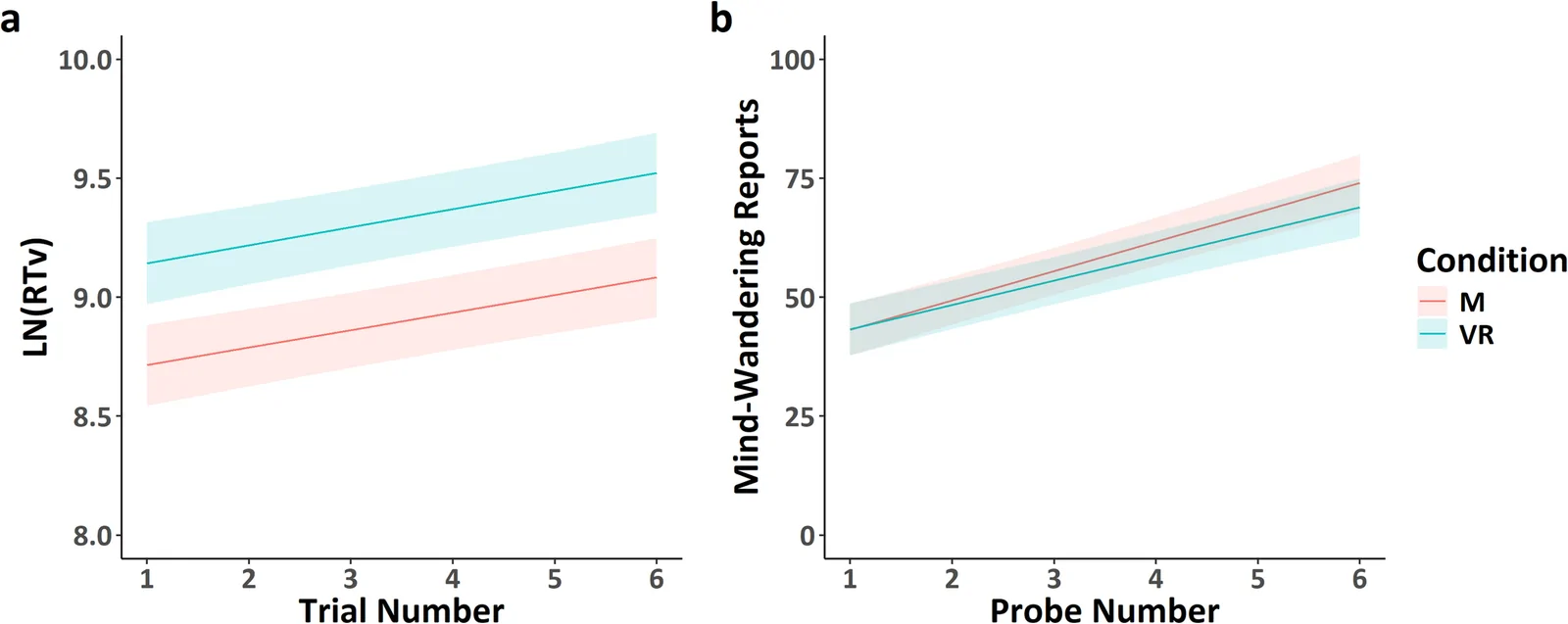
Predicted model outcomes for Model 1.1 and Model 1.2. Note: Panel 3a displays the predicted outcomes from Model 1.1, illustrating how task performance measured by LN(RTv) varies over time on task (i.e., trial number within Block 1) across experimental conditions. The plot compares performance under standard monitor-based (M) and virtual reality headset-based (VR) task presentation conditions. Higher LN(RTv) values indicate worse performance (i.e., greater response time variability). Panel 3b displays the predicted outcomes from Model 1.2, illustrating how mind-wandering reports varied over time on task (i.e. the probe number within Block 1) and across standard computer monitor (M) or virtual reality headset (VR) presentation conditions. Shaded ribbons represent 95% confidence intervals

We hypothesized that inattention, as indexed by response variability and mind-wandering, would be lower, and increase less aggressively over time on task when the task was presented with the VR HMD than on a standard monitor (Hypothesis 1). This was examined by modeling data from the first block of study (before some participants switched viewing devices) using Model 1.1 (response variability) and Model 1.2 (mind-wandering). The experimental block was divided into six equal segments, defined by evenly distributed probes with time operationalized as either the block segment (Model 2.1) or probe number (Model 2.2), the model outcomes are graphically depicted in Fig. 3a and b, respectively.

Model 1.1, which analyzed response variability (see Tables 3 and 4, Fig. 3a), revealed a significant increase over task segments (*b* = 0.07, 95% *CI* [0.05, 0.10], *SE* = 0.01, *p* < 0.001, *η*^2^ = 0.31), corresponding to approximately a 7% increase in variability per segment suggesting a performance decline throughout the course of the block. Contrary to our expectations, response variability was significantly higher in the VR condition than the monitor condition (*b* = 0.43, 95% *CI* [0.21, 0.68], *SE* = 0.11, *p* < 0.001, *η*^2^ = 0.08), representing approximately a 54% increase in variability and indicating worse performance in the VR condition. The interaction between viewing condition and block segment did not reach significance *(b* < 0.01, 95% *CI* [-0.03, 0.04], *SE* = 0.02, *p* = 0.897, *η*^2^ < 0.01).

**Table 3:** ANOVA tables for linear mixed effects models examining response time variability and mind-wandering

Model	Parameter	SS	MS	df_Num_	df_Den_	F	p	η2
1.1	Model Formula: ln(RTv) ~ condition*segment + (segment|participant) Model Data: Block 1, Observations = 15,516, Performance: *R*^*2*^_*Marginal*_ = 0.032, *R*^*2*^_*Conditional*_ = 0.302							
	condition	20.18	20.18	1	161.8	14.54	< 0.001	0.08
	segment	100.57	100.57	1	161.3	72.43	< 0.001	0.31
	condition:segment	0.02	0.02	1	161.3	0.02	0.898	< 0.01
1.2	Model Formula: MW ~ condition*probe + (probe|participant) Model Data: Block 1, Observations = 984, Performance: *R*^*2*^_*Marginal*_ = 0.108, *R*^*2*^_*Conditional*_ = 0.861							
	condition	60.02	60.02	1	162	0.49	0.486	< 0.01
	probe	22,156.37	22,156.37	1	162	179.68	< 0.001	0.53
	condition:probe	195.63	195.63	1	162	1.59	0.210	0.01
2.1	Model Formula: ln(RTv) ~ group*block*segment + (block|participant) Model Data: Both Blocks, Observations = 30,836, Performance: *R*^2^_Marginal_ = 0.026, *R*^2^_Conditional_ = 0.299							
	group	10.20	3.40	3	159.9	2.44	0.066	0.04
	block	7.92	7.92	1	159.7	5.69	0.018	0.03
	segment	481.33	481.33	1	30,510.8	345.82	< 0.001	0.01
	group:block	89.81	29.94	3	159.7	21.51	< 0.001	0.29
	group:segment	1.65	0.55	3	30,510.8	0.40	0.756	< 0.01
	block:segment	0.14	0.14	1	30,512.9	0.10	0.752	< 0.01
	group:block:segment	4.82	1.61	3	30,512.8	1.15	0.326	< 0.01
2.2	Model Formula: MW ~ group*block*probe + (probe + block|participant) Model Data: Both Blocks, Data: Observations = 1968, Performance: *R*^2^_Marginal_ = 0.161, *R*^2^_Conditional_ = 0.824							
	group	1394.01	464.67	3	160.0	3.14	0.027	0.06
	block	9663.99	9663.99	1	160.0	65.25	< 0.001	0.29
	probe	28,260.14	28,260.14	1	160.0	190.80	< 0.001	0.54
	group:block	1716.68	572.23	3	160.0	3.86	0.011	0.07
	group:probe	1583.17	527.72	3	160.0	3.56	0.016	0.06
	block:probe	5381.90	5381.90	1	1472.0	36.34	< 0.001	0.02
	group:block:probe	10,513.49	3504.50	3	1472.0	23.66	< 0.001	0.05

**Table 4:** Coefficient tables for linear mixed effects models examining response time variability and mind-wandering

Model	Predictors	b	Reverse Transformed	CI	SE	p
1.1	(Intercept)	8.90	7331.97 ms	8.74 – 9.06	0.08	< 0.001
	condition [VR]	0.43	+ 53.73%	0.21 – 0.66	0.11	< 0.001
	segment	0.07	+ 7.25%	0.05 – 0.10	0.01	< 0.001
	condition [VR] × segment	< 0.01	+ < 0.01%	-0.03 – 0.04	0.02	0.897
1.2	(Intercept)	58.57	-	53.64 – 63.50	2.51	< 0.001
	condition [VR]	-2.49	-	-9.50 – 4.52	3.57	0.486
	probe	6.17	-	5.01 – 7.33	0.59	< 0.001
	condition [VR] × probe	-1.06	-	-2.71 – 0.59	0.84	0.208
2.1	(Intercept)	8.89	7259.02 ms	8.67 – 9.12	0.11	< 0.001
	group [M-VR]	0.01	+ 1.01%	-0.31 – 0.32	0.16	0.964
	group [VR-M]	0.46	+ 58.41%	0.14 – 0.78	0.16	0.005
	group [VR-VR]	0.42	+ 52.20%	0.10 – 0.73	0.16	0.009
	block [block2]	0.11	+ 11.63%	0.00 – 0.21	0.05	0.043
	segment	0.08	+ 8.33%	0.06 – 0.10	0.01	< 0.001
	group [M-VR] × block [block2]	0.23	+ 25.86%	0.09 – 0.38	0.07	0.002
	group [VR-M] × block [block2]	-0.37	-30.93%	-0.52 – -0.22	0.08	< 0.001
	group [VR-VR] × block [block2]	-0.03	-2.96%	-0.17 – 0.11	0.07	0.681
	group [M-VR] × segment	-0.01	-1.00%	-0.04 – 0.02	0.02	0.595
	group [VR-M] × segment	-0.02	-1.98%	-0.05 – 0.01	0.02	0.310
	group [VR-VR] × segment	0.01	+ 1.01%	-0.02 – 0.04	0.02	0.460
	block [block2] × segment	< -0.00	+ < 0.01%	-0.03 – 0.03	0.02	0.813
	(group [M-VR] × block [block2]) × segment	0.02	+ 2.02%	-0.03 – 0.06	0.02	0.493
	(group [VR-M] × block [block2]) × segment	0.01	+ 1.01%	-0.03 – 0.06	0.02	0.599
	(group [VR-VR] × block [block2]) × segment	-0.02	-1.98%	-0.07 – 0.02	0.02	0.313
2.2	(Intercept)	62.12	-	55.20 – 69.04	3.53	< 0.001
	group [M-VR]	-7.18	-	-17.02 – 2.67	5.02	0.153
	group [VR-M]	-6.26	-	-16.23 – 3.71	5.09	0.218
	group [VR-VR]	-5.83	-	-15.62 – 3.96	4.99	0.243
	block [block2]	16.48	-	11.17 – 21.80	2.71	< 0.001
	probe	6.36	-	4.91 – 7.81	0.74	< 0.001
	group [M-VR] × block [block2]	-11.62	-	-19.19 – -4.05	3.86	0.003
	group [VR-M] × block [block2]	-8.02	-	-15.68 – -0.35	3.91	0.040
	group [VR-VR] × block [block2]	-1.94	-	-9.46 – 5.58	3.83	0.612
	group [M-VR] × probe	-0.38	-	-2.44 – 1.69	1.05	0.721
	group [VR-M] × probe	-1.43	-	-3.52 – 0.66	1.07	0.181
	group [VR-VR] × probe	-1.08	-	-3.13 – 0.97	1.05	0.303
	block [block2] × probe	-5.42	-	-6.66 – -4.17	0.63	< 0.001
	(group [M-VR] × block [block2]) × probe	5.15	-	3.38 – 6.92	0.90	< 0.001
	(group [VR-M] × block [block2]) × probe	6.93	-	5.14 – 8.73	0.91	< 0.001
	(group [VR-VR] × block [block2]) × probe	1.83	-	0.07 – 3.59	0.90	0.042

To aid interpretation of response time variability coefficients in Model 1.1 and Model 2.1 we also present the reverse transformed model intercepts and relative influence of the predictors

With regards to mind-wandering, Model 1.2 revealed (see Table 3 and 4, Fig. 3b) that mind-wandering increased significantly over successive probes (*b* = 6.17, 95% *CI* [5.01, 7.33], *SE* = 0.59, *p* < 0.001, *η*^2^ = 0.53), indicating an average increase of approximately 6.17 percentage points per probe. However, the rate of increase did not vary significantly between those who viewed the stimuli on a monitor and those who viewed them in VR (*b* = -1.06, 95% *CI* [-2.71, 0.59], *SE* = 0.84, *p* = 0.208, *η*^2^ < 0.01). There was no overall difference in mind-wandering between the two display conditions (*b* = -1.06, 95% *CI* [-2.71, 0.59], *SE* = 0.84, *p* = 0.208, *η*^2^ = 0.01).

### Switching versus Not switching device type

**Fig. 4:**
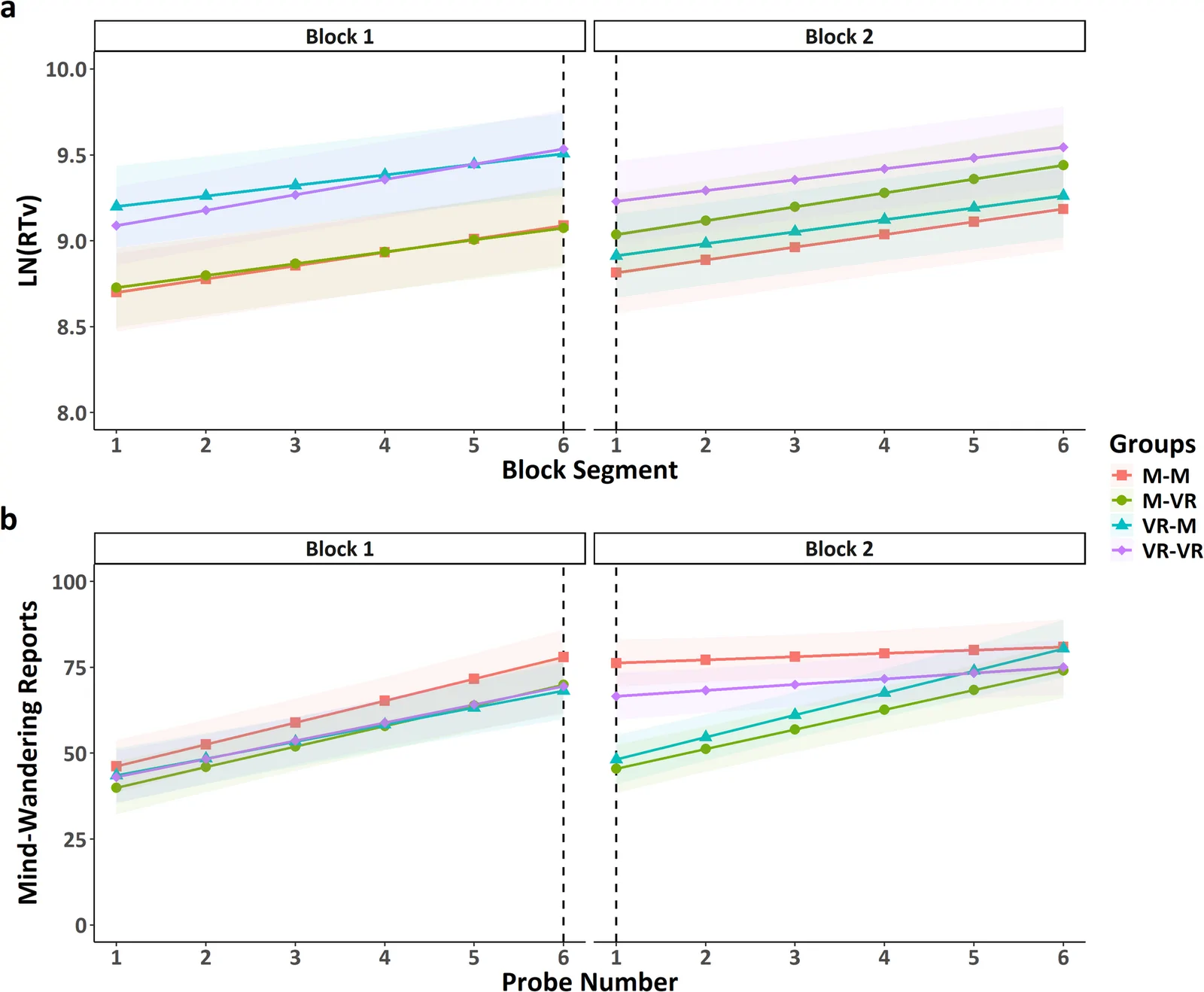
Predicted model outcomes for Model 2.1 and Model 2.2. Note: Participant group names (M-M, M-VR, VR-M, and VR-VR) indicate the order of Monitor (M) and Virtual Reality (VR) conditions, with the first initial representing the condition of the group’s first block and the second initial the condition of the group’s second block. Panel 4a displays the predicted outcomes from Model 2.1, illustrating how task performance. measured by LN(RTv), varies over time on task (i.e., block segment), between experimental blocks, and across experimental groups. Higher LN(RTv) values indicate worse performance (i.e., greater response time variability). Panel 4b displays the predicted outcomes from Model 1.2, illustrating how mind-wandering reports varied over time on task (i.e. block probe number), between experimental blocks and across experimental groups. The dashed vertical lines at the end of Block 1 and the Start of Block 2 in both panels indicate the point of comparison used for testing Hypothesis 2 and Hypothesis 3. Shaded ribbons represent 95% confidence intervals

The second hypothesis was that switching to a distinct device would lead to greater attention and improved performance compared to continuing with the same device in the second half (i.e., block) of the study. To test this, we modeled data from the full experiment using Model 2.1, which focused on the measure of response variability (see Table 3 and 4, Fig. 4a), and Model 2.2, which focused on mind-wandering (see Table 3 and 4, Fig. 4b). Each experimental block was divided into six equal segments, defined by evenly distributed probes. Consequently, time was operationalized as either the block segment (Model 2.1) or probe number (Model 2.2).

**Fig. 5:**
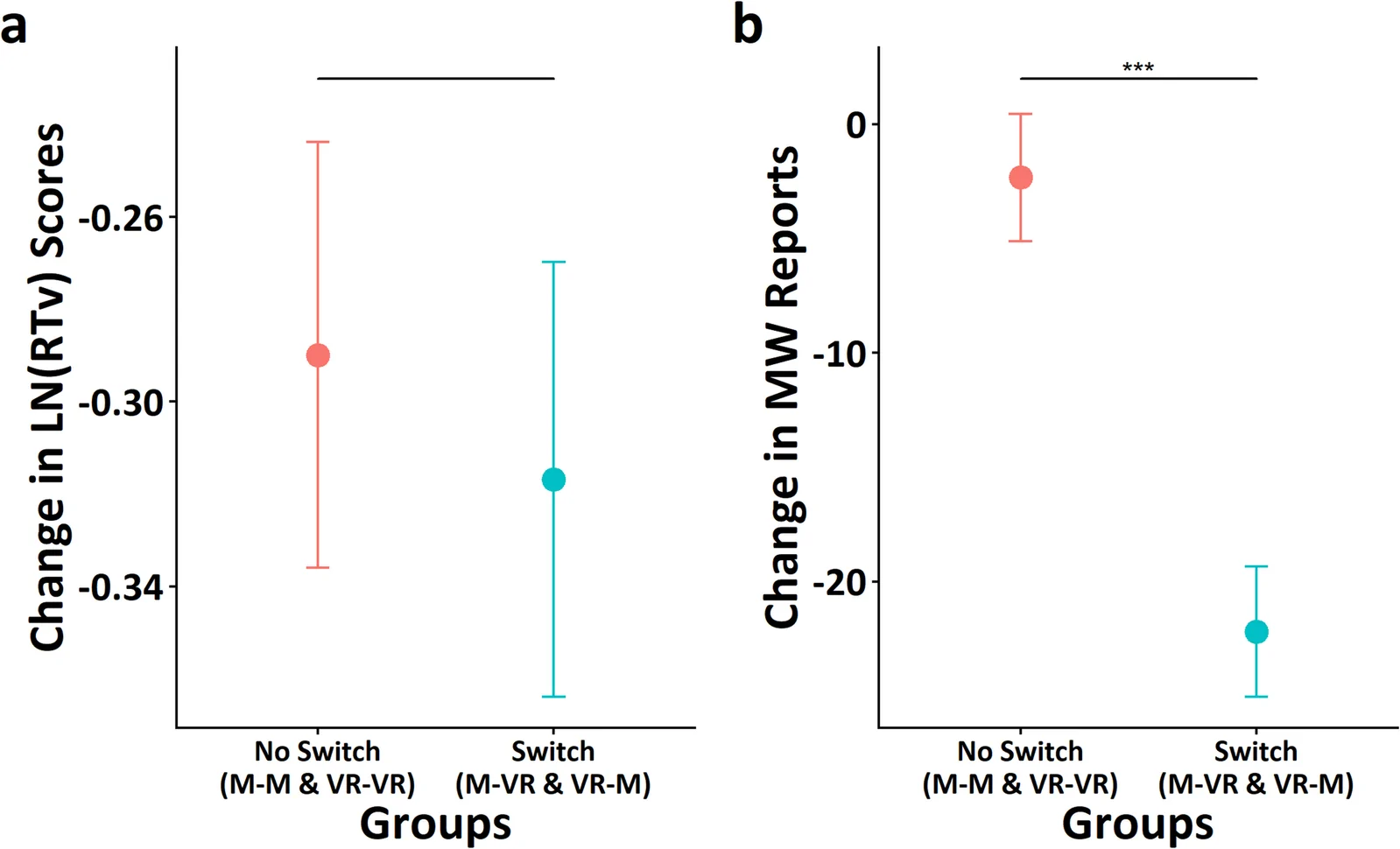
The effect of switching task presentation condition on task performance and mind-wandering. Note: Participant group names (M-M, M-VR, VR-M, and VR-VR) indicate the order of conditions Monitor (M) and Virtual Reality (VR) with the first initial representing the condition of the group’s first block and the second initial the condition of the group’s second block. Panel 5a displays the difference in the estimated marginal means in Model 2.1 of response time variability scores from the end of Block 1 to the start of Block 2 between groups that switched or did not switch their task presentation condition. Panel 5b displays the difference in the estimated marginal means in Model 2.2 of mind-wandering reports from the end of Block 1 to the start of Block 2 between groups that switched or did not switch their task presentation condition. Error bars represent ± 1 SE. Pairwise comparisons with significance scores of the contrasts are shown at the top, (* = *p* < 0.05, ** = *p* < 0.01, *** = *p* < .001)

To directly address the hypothesis and examine the behavioural change between the blocks, we used contrasts to compare the marginal means between the end of Block 1 (i.e., Segment/Probe 6 in Block 1) and the start of Block 2 (i.e., Segment/Probe 1 in Block 2) for groups that switched devices (M–VR, VR–M) versus those that remained on the same device (M–M, VR–VR). In Model 2.1, this comparison did not reveal a significant change in task performance between switch and no-switch groups, *z* = -0.41, *p* = 0.683, *d* = -0.02 (see Fig. 5a). However, consistent with our hypothesis, Model 2.2 revealed a very large and statistically significant reduction in mind-wandering in participants who switched devices (*M* = -22.18, *SE* = 2.85) compared to those who did not (*M* = -2.33, *SE* = 2.78), *t*(160) = -4.98, *p* < 0.001, *d* = -1.63 (see Fig. 5b).

### Switching monitor to VR versus VR to monitor

Finally, we evaluated whether transitioning from Monitor to VR would lead to greater restoration of attention and performance than transitioning from VR to Monitor (Hypothesis 3). As with Hypothesis 2, this analysis focused on specific contrasts within the full dataset modelled using Model 2.1 measuring response variability (see Table 3 and 4, Fig. 4a), and Model 2.2 measuring mind-wandering (see Table 3 and 4, Fig. 4b).

**Fig. 6:**
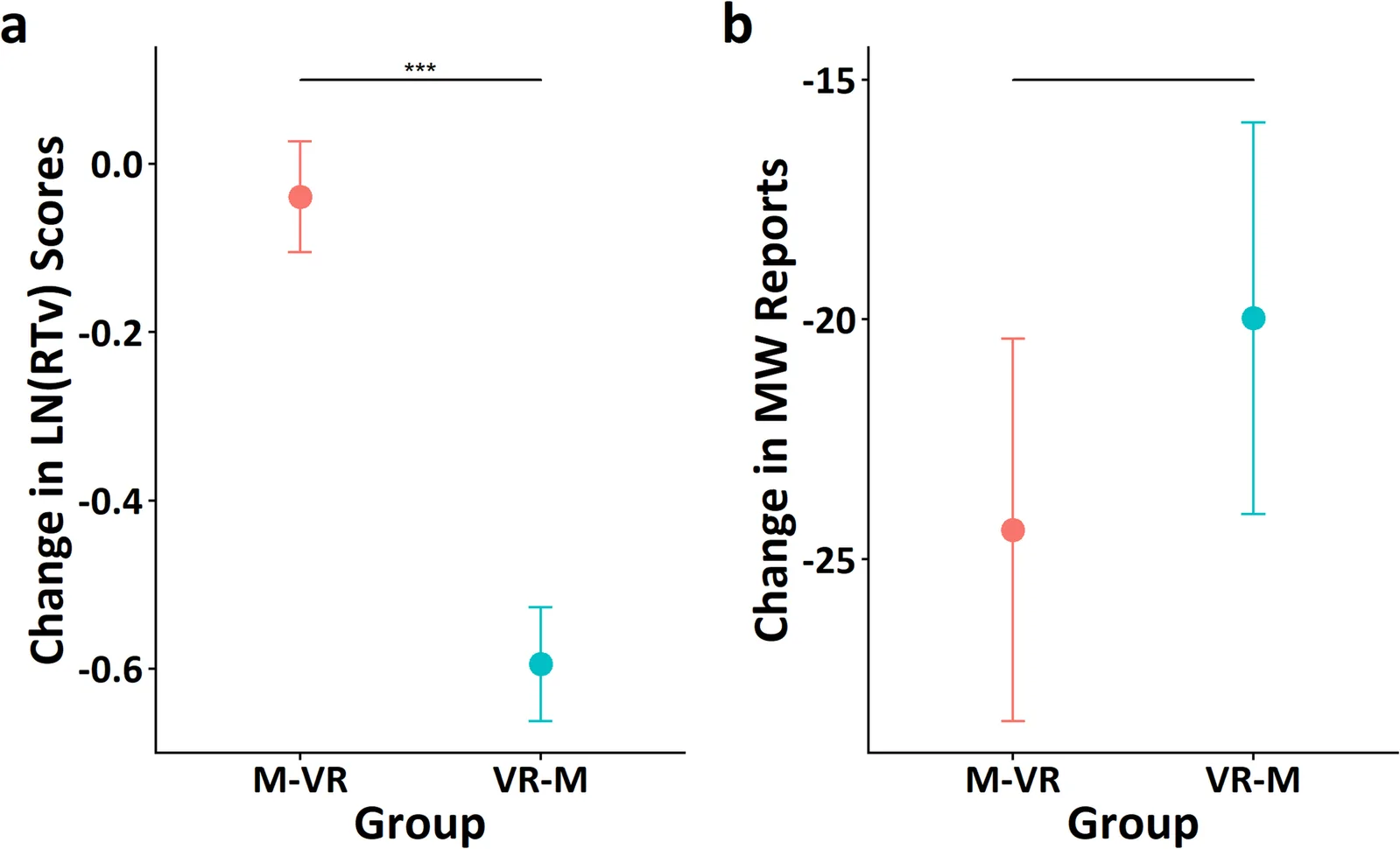
How different presentation condition switches influence task performance and mind-wandering. Note: Panel 6a displays the difference in the estimated marginal means in Model 2.1 of response time variability scores from the end of Block 1 to the start of Block 2 between the different switch conditions. Panel 6b displays the difference in the estimated marginal means in Model 2.2 of mind-wandering reports from the end of Block 1 to the start of Block 2 between the different switch conditions. Error bars represent ± 1 SE. Comparison with significance scores of the contrasts are shown at the top, (* = *p* < 0.05, ** = *p* < 0.01, *** = *p* < .001)

To test Hypothesis 3, we contrasted changes in behaviour from the end of Block 1 (i.e., Segment/Probe 6 in Block 1) to the start of Block 2 (i.e., Segment/Probe 1 in Block 2) between the two device-switching groups: those who transitioned from Monitor to VR (M–VR) and those who transitioned from VR to Monitor (VR–M). In Model 2.1, this contrast revealed a statistically significant difference in performance changes across blocks between the two groups, with an approximately 45% reduction in response variability in the VR–M group (*M* = -0.59, *SE* = 0.07), compared to an approximately 4% reduction in response variability in the M–VR group (*M* = -0.04, *SE* = 0.07), *z* = 5.89, *p* < 0.001, *d* = 0.05 (see Fig. 6a), indicating greater performance restoration in the VR–M group. However, the same contrast applied to Model 2.2 did not yield a significant difference in the reduction of mind-wandering, *t*(160) = -0.77, *p* = 0.441, *d* = -0.36 (see Fig. 6b), suggesting that attention, as measured through reported mind-wandering, was not differentially affected by the direction of the device switch.

## Discussion

The present study yielded several main findings. The first set of notable findings pertain to attentional engagement and performance in the first block of trials. In line with expectations, as time unfolded in the first half of the study, performance progressively decreased and mind-wandering increased, indicating a measurable sustained attention decrement. While the performance decline was the same when the visual metronome response task (VMRT) was presented in VR compared to a standard computer monitor, overall performance was poorer in the VR condition. This aligns with the "novelty effect" proposed by Miguel-Alonso et al. (2024), wherein the unfamiliar aspects of the VR environment may have imposed additional cognitive processing demands that interfered with task performance. The pattern of mind-wandering reports in the first half of the experiment was more nuanced. Although there was no overall difference in mind-wandering between VR and monitor conditions, participants in the monitor condition showed a slightly greater increase in mind-wandering over time compared to those in VR. This suggests that while VR may have impaired performance through increased cognitive load, it may have simultaneously helped maintain some degree of task engagement, potentially through its new and immersive qualities. Diagnostic analyses examining stimulus presentation and response recording precision between setups (available in the analysis script in the OSF repository) indicate comparable measurement accuracy across conditions, making it unlikely that technical factors account for the observed performance differences.

Having shown a sustained attention decrement in the first half of the experiment, we also demonstrated that mind-wandering is impacted substantially by a context switch. Specifically, participants who switched between presentation modes (either Monitor to VR or VR to Monitor) showed a large reduction (*d* = -1.63) in mind-wandering compared to those who continued with the same device. By introducing a different presentation context while keeping the task unchanged, participants appear to have experienced a form of subjective dishabituation that re-engaged their attention to the task (see Giambra 1995; Smallwood et al. 2004; Thomson et al. 2015a, b). However, while the context switch across VR and monitor (relative to no switch) had a large impact on mind-wandering, it did not have a measurable influence on performance in the VMRT.

This dissociation between mind-wandering and performance effects deserves careful consideration given the substantial body of research establishing robust links between increased mind-wandering and decreased performance. Most directly relevant to the present study, previous research using the metronome response task has consistently found that increases in mind-wandering are associated with increases in response time variability (Anderson et al. 2021; Laflamme et al. 2018; Safati et al. 2024; Seli et al. 2013). This relationship extends beyond the MRT, with meta-analytic evidence indicating that mind-wandering predicts performance errors across a variety of laboratory tasks (Randall et al. 2014) and is broadly associated with lapses of attention in sustained attention paradigms (Unsworth & Robison 2016). We acknowledge that the mind-wandering-performance relationship is not invariant and can be moderated by factors such as individual differences in executive control (Kane & McVay 2012), task demands (Franklin et al. 2014), and whether mind-wandering is intentional or unintentional (Seli et al. 2016). In our study, the dissociation between mind-wandering and performance effects following the context switch may reflect that the performance costs associated with VR masked performance improvements even as participants experienced renewed engagement with the task.

The dissociation we observed also raises broader questions about the nature of mind-wandering reports themselves. While numerous studies have documented robust associations between mind-wandering and performance decrements (as reviewed above), recent experimental work suggests that mind-wandering reports may, in some paradigms, be inferred from performance (Esau & Helton 2025; Polychroni et al. 2025), underscoring that the direction of the relationship between subjective reports and objective measures should not be assumed. For example, Polychroni et al. (2025) demonstrated that false performance feedback can systematically influence participants’ retrospective reports of mind-wandering, with participants reporting more mind-wandering when led to believe their performance was poor, even when their actual performance was unchanged. Our finding of reduced mind-wandering reports following context switches, despite no corresponding performance improvements, is inconsistent with the notion that participants simply infer their attentional state from their recent task performance. Instead, it suggests that the context switch may have genuinely altered participants’ subjective experience of task engagement, independent of objective performance. The mechanisms underlying this dissociation remain unclear and represent an important direction for future research into the relationship between subjective attentional states and objective performance measures.

Finally, we were able to compare how performance and attention changed specifically when the task context switched from VR to a monitor and vice versa. While participants switching from VR to the 2D monitor showed slightly better performance restoration than those switching from the monitor to VR, this effect was small (*d* = 0.05). This finding is somewhat unexpected. However, it may again be the result of the ‘novelty effect’ of distracting stimuli, leading to poorer performance in the VR condition regardless of whether it was completed before or after the switch. We also note that this small effect on performance was not accompanied by corresponding changes in mind-wandering. This suggests that the attentional benefits of context switching are driven primarily by the change in presentation environment itself, rather than the specific characteristics of the contexts being switched between.

These findings suggest that introducing contextual variability may represent a practical and easily implemented intervention for combating experiences of attentional decline in sustained tasks, though the optimal timing and magnitude of such changes requires further investigation. Despite these promising findings, several limitations should be acknowledged. First, our sample consisted primarily of undergraduate students who may have had limited experience with VR technology. The poorer overall performance observed in VR relative to when the task was presented on a 2-D monitor might be reduced or eliminated with more experienced users. Second, we did not directly measure potential factors that might mediate attentional declines or the effects of context switches. For example, previous research has shown that factors such as presence (e.g., Maneuvrier et al. 2020), cognitive load (for a review see Han et al. 2021), or physical comfort (e.g., Mimnaugh et al. 2023) are relevant to studies within VR environments. Third, future research could examine the duration and generalizability of the context-switching effect we have observed. For instance, it would be useful to explore whether switching between other immersive or non-immersive contexts (e.g., different VR environments or physical room changes) produces similar effects (cf. Huang et al. 2021). Additionally, longitudinal designs could help determine whether the effects of context switching persist with repeated exposures or diminish as new context effects wear off. Finally, disentangling the interaction between environmental variability and other known modulators of attention, such as motivation (Brosowsky et al. 2023) and task difficulty (Brosowsky et al. 2021), could help build a more comprehensive understanding of how attentional resources are deployed and maintained over time.

## Supplementary Information

Below is the link to the electronic supplementary material.

Supplementary file1 (DOCX 18 kb)

## Data Availability

The deidentified experimental data, analysis code, and experimental materials are available in an OSF repository https://osf.io/qymw3/?view_only=ebeac74209f845baa0b52d0aa1ea31be.
