# Supplementary material for: Switching between a computer monitor and virtual reality to reduce the sustained attention decrement
Source: Virtual Real. 2026 Aug 9;30(4):200. doi: 10.1007/s10055-026-01424-0 (PMC13740080; doi:10.1007/s10055-026-01424-0)
Supplement: Supplementary file 1 [file 10055_2026_1424_MOESM1_ESM.docx]

**Table S1 Comparison of linear mixed-effects model random effects specifications**

| Comparison | Name | Formula | AIC | R2_conditional | R2_marginal | RMSE |
| --- | --- | --- | --- | --- | --- | --- |
| **RTV Block 1** | | | | | | |
| RTV Block 1 | Model 1.1 | LNRRTv ~ condition * sub_block + (sub_block \| participant) | 49876.896 | 0.302 | 0.032 | 1.169 |
| RTV Block 1 | Model 1.1a | LNRRTv ~ condition * sub_block + (1 \| participant) | 49963.749 | 0.291 | 0.032 | 1.181 |
| **Probe Block 1** | | | | | | |
| Probe Block 1 | Model 1.2 | response ~ condition * probe_number + (probe_number \| participant) | 8302.765 | 0.843 | 0.122 | 9.382 |
| Probe Block 1 | Model 1.2a | response ~ condition * probe_number + (1 \| participant) | 8463.683 | 0.746 | 0.122 | 12.960 |
| **RTV Full** | | | | | | |
| RTV Full | Model 2.1 | LNRRTv ~ group * block * sub_block + (block \| participant) | 98648.043 | 0.299 | 0.026 | 1.174 |
| RTV Full | Model 2.1a | LNRRTv ~ group * block * sub_block + (1 \| participant) | 98876.036 | 0.288 | 0.026 | 1.185 |
| **Probe Full** | | | | | | |
| Probe Full | Model 2.2 | response ~ group * block * probe_number + (probe_number + block \| participant) | 16562.407 | 0.814 | 0.170 | 10.772 |
| Probe Full | Model 2.2a | response ~ group * block * probe_number + (probe_number \| participant) | 16943.701 | 0.717 | 0.171 | 13.904 |
| Probe Full | Model 2.2b | response ~ group * block * probe_number + (1 \| participant) | 17052.673 | 0.668 | 0.171 | 15.507 |
